# Burden of intestinal protozoan parasites coinciding with *Helicobacter pylori* infection in patients with gastrointestinal symptoms

**DOI:** 10.1186/s13099-026-00841-5

**Published:** 2026-06-07

**Authors:** Amel Youssef Shehab, Mona Mohamed Tolba, Ali Ali Mohammed Alrabeei, Heba S. Ibrahim, Hend El-Taweel

**Affiliations:** 1 Department of Parasitology, Medical Research Institute, Alexandria University, Alexandria, Egypt; 2 Department of Medical Laboratories, Almahweet University, Al-Mahwit city, Yemen

**Keywords:** Stool examination, Intestinal protozoa, *Blastocystis* spp., *Helicobacter pylori*, Coproantigen, Coinfections, Gastrointestinal symptoms

## Abstract

**Aim:**

Protozoal infections are underdiagnosed contributors to gastrointestinal disease. As their symptoms frequently overlap with those of *Helicobacter pylori* infection, a leading cause of gastrointestinal illness, coinfections may be overlooked. This study aimed to determine the prevalence of intestinal protozoal infections among patients referred for *H. pylori* stool testing and to explore their association with clinical symptoms and risk factors.

**Methods:**

A cross-sectional study was conducted on 150 patients presenting with gastrointestinal complaints. Stool samples were examined using comprehensive parasitological techniques, along with ELISA for *Entamoeba histolytica/dispar* and *H. pylori* antigens.

**Results:**

Intestinal protozoa were detected in 36.9% of participants, most commonly *Blastocystis* spp. (33.3%), followed by *E. dispar* (5.3%) and *E. coli* (2.7%). *Giardia lamblia*,* Dientamoeba fragilis*, and *Cyclospora cayetanensis* were detected in only four patients. All *Entamoeba* infections were negative for *E. histolytica-*specific antigen. *H. pylori* antigen was positive in 35.3% of participants. The prevalence of intestinal protozoa did not differ significantly between *H. pylori*-positive and *H. pylori*-negative patients (39.6% vs. 34%); however, *Blastocystis* spp. was significantly associated with higher *H. pylori* antigen concentrations. Both protozoal and *H. pylori* infections were significantly associated with the source of drinking water but showed no significant association with age or sex. Abdominal pain was the predominant symptom among all patients, regardless of *H. pylori* or protozoal infection status. Notably, 78% of patients referred for *H. pylori* testing had not concurrently undergone parasitological examination, of whom 37.6% were subsequently found to have protozoal infections.

**Conclusion:**

This study demonstrates a high prevalence of intestinal protozoal infections, predominantly *Blastocystis* spp., among patients undergoing *H. pylori* evaluation, and identifies a significant association between *Blastocystis* and elevated *H. pylori* antigen levels. The overlapping symptoms and frequent underdiagnosis of protozoal infections in clinical practice support the incorporation of routine parasitological examination alongside *H. pylori* testing in patients presenting with gastrointestinal symptoms, with the potential to improve diagnostic accuracy and patient management.

## Introduction

Gastrointestinal illness remains one of the most common health problems worldwide, with a significant proportion attributable to intestinal protozoa. These unicellular microparasites inhabit the intestinal tract of humans and other vertebrates, contributing substantially to global morbidity [1, 2]. Their impact is particularly pronounced in low- and middle-income countries, where contaminated water and food facilitate transmission [3]; however, they are increasingly recognized as important causes of disease even in high-income settings [4].

The spectrum of common protozoan infections includes *Giardia lamblia*, *Blastocystis* spp., *Dientamoeba fragilis*, *Cryptosporidium* spp., *Cyclospora cayetanensis*, and *Entamoeba histolytica* [5, 6]. These organisms may cause nonspecific symptoms, such as diarrhea, abdominal pain, bloating, and weight loss, or remain entirely asymptomatic. Because they often mimic bacterial, viral, or functional gastrointestinal disorders, they are frequently underrecognized in clinical practice [7]. Moreover, standard diagnostic routines prioritize bacterial and viral pathogens, leaving protozoal infections both inadequately detected and poorly documented [8], and many patients with persistent gastrointestinal symptoms of protozoal etiology consequently remain unidentified.

Even when suspected, intestinal protozoa can be challenging to confirm. Routine microscopy, although widely used, cannot reliably differentiate pathogenic from non-pathogenic *Entamoeba* species and may fail to detect coccidian parasites without specialized stains [9–11]. Clinical laboratories often face additional constraints, including incomplete clinical history, limited clinician awareness, and inadequate diagnostic resources, all of which contribute to missed diagnoses and ongoing transmission [12].

*Helicobacter pylori (H. pylori)* is aGram-negative bacterium and one of the most prevalent chronic infections worldwide, colonizing the stomach of nearly half the global population [13, 14]. Although frequently asymptomatic, it is a major cause of chronic dyspepsia, gastritis, and peptic ulcer disease [15, 16]. Complicated *H. pylori* infection may present with alarm features such as gastrointestinal bleeding, melena, hematemesis, dysphagia, or unintentional weight loss, whereas uncomplicated infection often manifests with nonspecific symptoms, including abdominal pain, bloating, nausea, and dyspepsia, that overlap with those of intestinal protozoal infections [7, 15, 16]. As both infections may reach substantial levels in endemic settings [2, 13, 14], individuals living in such areas may be simultaneously exposed to both pathogens [17]. Despite this, the true frequency of coinfection and its clinical relevance remain poorly understood, as systematic co-evaluation of both infections is rarely performed in routine practice. Diagnostic approaches that focus on a single pathogen not only risk missing concurrent infections but may also lead to incomplete treatment and persistent symptoms. This study, therefore, aimed to identify intestinal protozoal infections among patients presenting with gastrointestinal symptoms and undergoing evaluation for *H. pylori*, and to assess potential associations, supporting more comprehensive management of gastrointestinal disease.

## Materials and methods

### Study design and participants

A cross-sectional study was conducted between January and October 2024 on 150 patients of both sexes presenting with gastrointestinal symptoms, referred to the Parasitology Department Laboratory, Medical Research Institute, Alexandria University, for *H. pylori* evaluation. Patients who had received antibiotics, antiparasitic, or anti-ulcer therapy within the preceding month, those who were immunocompromised, or those with a prior diagnosis of *H. pylori*-related complications or other chronic gastrointestinal disorders were excluded. Demographic and clinical data, including age, sex, residence, symptoms, and risk factors (animal contact, eating habits, and drinking water source), were collected using a structured questionnaire.

### Ethical considerations

Ethical approval was granted by the Research Ethics Committee of the Medical Research Institute (IORG008812), Alexandria University. Informed consent was obtained from all participants or their guardians prior to enrollment.

### Sample collection and preservation

A single fresh stool sample was collected from each participant, homogenized, and divided into three aliquots: the first was examined immediately for intestinal protozoa; the second was stored at -20 °C for *E. histolytica/dispar* coproantigen testing; and the third was stored at -20 °C for *H. pylori* antigen detection and quantification.

### Parasitological examination

Stool samples were examined using direct wet mount microscopy in saline and iodine at 10× and 40× magnification, followed by formalin-ethyl acetate sedimentation to enhance detection sensitivity [18]. Permanent smears were stained with trichrome stain for identification of protozoan cysts and trophozoites, whereas modified Ziehl-Neelsen staining was used for detection of coccidian oocysts [19].

### Antigen detection

Stool samples were initially screened for *E. histolytica/dispar* coproantigen using a commercial ELISA kit (Diagnostic Automation Inc.^®^). Positive samples were subsequently retested with the DRG Diagnostics^®^ *E. histolytica*-specific coproantigen ELISA to confirm the presence of pathogenic *E. histolytica*. Quantitative *H. pylori* stool antigen detection was performed using the Quantitative *H. pylori* Ag ELISA kit (Creative Diagnostics, Microwell ELISA Diagnostic Systems, USA) according to the manufacturer’s protocol. This assay has a reported diagnostic sensitivity of 98.6% [20]. Optical density was measured at 450 nm, and results were interpreted as negative (< 15 ng/ml), equivocal (15–20 ng/ml), or positive (> 20 ng/ml). Positive results were further categorized as high (> 70 ng/ml) or low (< 70 ng/ml) concentration.

### Statistical analysis

Data were analyzed using IBM SPSS Statistics version 20.0 (Armonk, NY, USA). Categorical variables are presented as frequencies and percentages; continuous variables are expressed as mean ± standard deviation (SD). Associations between categorical variables were assessed using the Chi-square test or Fisher’s exact test, as appropriate. A *p*-value ≤ 0.05 was considered statistically significant.

## Results

### Participant characteristics

Participants ranged in age from 8 to 70 years (mean ± SD: 30.12 ± 13.83), with the majority (58%) aged between 21 and 45 years. Females accounted for 58.7% of the sample, and 98.7% of participants resided in urban areas.

### Detection of intestinal protozoa and *H. pylori*

**Fig. 1:**
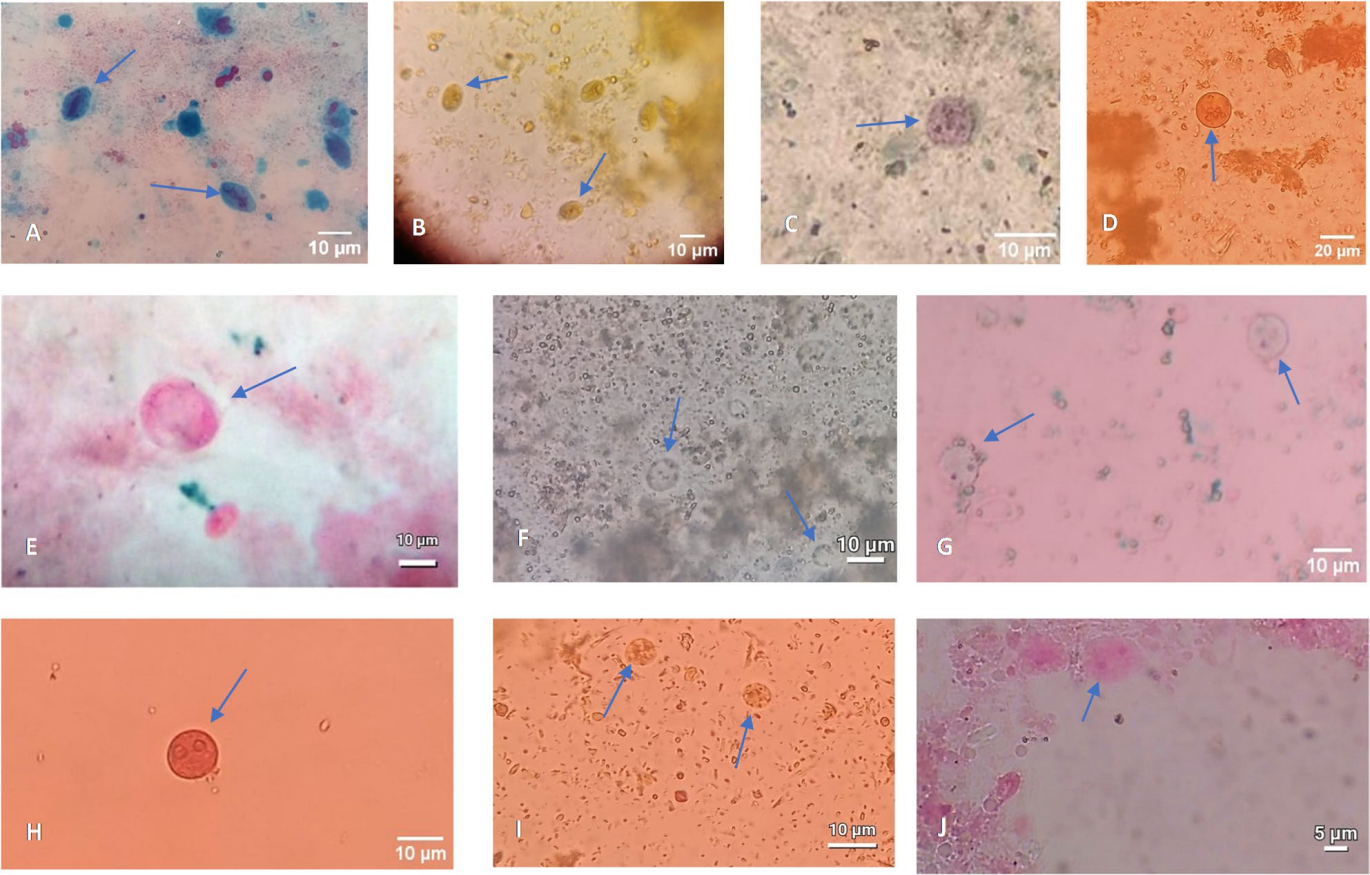
Intestinal protozoa detected in stool samples of participants. **(A–B)**: *G. lamblia* cysts stained with trichrome (×1000) and iodine wet mount (×400) **(C)**: *C. cayetanensis* oocyst stained with modified Ziehl–Neelsen (×1000) **(D)**: *E. coli* cyst in iodine wet mount (×400) (**E–F)**: *Blastocystis* spp. vacuolar forms in trichrome stain (×1000) and saline wet mount (×400) **(G–H)**: *E.a histolytica/dispar* cysts in trichrome stain (×1000) and iodine wet mount (×400) (**I–J)**: *D. fragilis* trophozoites in iodine wet mount (×400) and trichrome stain (×1000)

Of the 150 participants examined, 54 (36.9%) were positive for intestinal protozoa; most harbored single infections (30%), while 4.7% had double and 1.3% had triple infections. *Blastocystis* spp. was the most frequently detected protozoan (33.3%), followed by *E. dispar* (5.3%) and *E. coli* (2.7%). *G. lamblia*,* D. fragilis*, and *C. cayetanensis* were uncommon, detected in only four patients (Fig. 1). No helminthic infections were detected. All infections initially identified as *E. histolytica/dispar* by microscopy or non-specific ELISA tested negative for *E. histolytica*-specific antigen, indicating that the detected species were likely nonpathogenic. *H. pylori* antigen was positive in 53 participants (35.3%).

### Coinfection patterns of intestinal protozoa and *H. pylori*

Intestinal protozoa were detected more frequently among *H. pylori*-positive participants than among *H. pylori*-negatives (39.6% vs. 34%), though this difference did not reach statistical significance. *Blastocystis* spp. and *E. dispar* were detected in 35.8% and 9.4% of *H. pylori*-positive individuals, respectively, compared with 32.0% and 3.1% of *H. pylori*-negative individuals (Table 1).

**Table 1:** Association between intestinal protozoa and *H. pylori* infections

(IPI)	*H. pylori*				χ^2^	*p*
	Positive(n=53)		Negative(n=97)			
	No.	(%)	No.	(%)		
Intestinal protozoa						
*** Blastocystis *** **spp.**						
Positive	19	(35.8)	31	(32.0)	0.233	0.629
Negative	34	(64.2)	66	(68.0)		
*** D. fragilis***						
Positive	0	(0.0)	2	(2.1)	1.108	^FE^*p*=0.540
Negative	53	(100.0)	95	(97.9)		
*** C. cayetanensis***						
Positive	0	(0.0)	1	(1.0)	0.550	^FE^*p*=1.000
Negative	53	(100.0)	96	(99.0)		
*** G. lamblia***						
Positive	1	(1.9)	0	(0.0)	1.842	^FE^*p*=0.353
Negative	52	(98.1)	97	(100.0)		
*** E. dispar***						
Positive	5	(9.4)	3	(3.1)	2.730	^FE^*p*=0.131
Negative	48	(90.5)	94	(96.9)		
*** E. coli***						
Positive	1	(1.9)	3	(3.1)	0.192	^FE^*p*=1.000
Negative	52	(98.9)	94	(96.9)		
**Total**						
Positive	2132	(39.6)	33	(34.0)	0.467	0.494
Negative	32	(60.4)	64	(66.0)		

χ2, p: χ2 and p values for Chi square test

^FE^*p*: *p*value for Fisher Exact

A significant association was observed between high *H. pylori* coproantigen levels and protozoal infection overall (54.3% vs. 15.0%, *p* = 0.011), with the strongest association observed for *Blastocystis* spp. (54.5% vs. 5.0%, *p* < 0.001) (Table 2).

**Table 2:** Association between intestinal protozoa and *H. pylori* coproantigen level

Parasitic infection	H. pylori coproantigen level				^χ2^	*p*
	High (*n*=33)		Low (n = 20)			
	No.	(%)	No.	(%)		
**Positive**	18	(54.5)	3	(15.0)	8.919	0.011^*^
* Blastocystis*spp.	18	(54.5)	1	(5.0)	13.292	<0.001^*^
* E. dispar*	3	(9.1)	2	(10.0)	0.012	1.000
* E. coli*	1	(3.0)	0	(0.0)	0.618	1.000
* G. lamblia*	1	(3.0)	0	(0.0)	0.618	1.000

χ^2^, *p*: χ^2^ and *p* values for Chi square test for comparing between the two groups

High concentration: > 70 ng/ml

Low concentration: < 70 ng/ml

*Statistically significant at p<0.05

### Gastrointestinal symptoms in relation to intestinal protozoa and *H. pylori*

Abdominal pain was the most common symptom across all infection groups (81.8–96.9%). Among patients with intestinal protozoal infection alone, diarrhea (48.5%) and loss of appetite (45.5%) were the next most frequent symptoms, whereas in *H. pylori*-only infection, loss of appetite (68.8%) and nausea (62.5%) predominated. Coinfected patients most frequently reported nausea and diarrhea (52.4% each). Notably, 64 participants tested negative for both infections, yet still experienced gastrointestinal symptoms, including abdominal pain, nausea, and diarrhea. With respect to symptom duration, patients with *H. pylori* infection, whether alone or in combination with protozoal infection, were significantly more likely to report symptoms lasting more than three months compared with uninfected participants (*p* = 0.006) (Table 3).

**Table 3:** Gastrointestinal symptoms in patients with intestinal protozoal and/or *H. pylori* infection

GIT symptoms	All negative (*n* = 64)		IPI alone (*n* = 33)		H. *p* alone (*n* = 32)		Both IPI and H. *p* (*n* = 21)		*p*
	No.	(%)	No.	(%)	No.	(%)	No.	(%)	
**Abdominal pain**	53	82.8	27	81.8	31	96.9	19	90.5	0.169
**Diarrhea**	21	32.8	16	48.5	13	40.6	11	52.4	0.300
**Vomiting**	22	34.4	7	21.2	10	31.3	8	38.1	0.514
**Nausea**	35	54.7	14	42.4	20	62.5	11	52.4	0.438
**Loss of appetite**	31	48.4	15	45.5	22	68.8	9	42.9	0.160
**Symptoms > 3 months**	24	37.5	19	57.6	22	68.8^**^	15	71.4^**^	0.006^*^

IPI: Intestinal protozoal infection

*H. p*: *Helicobacter pylori*

*p*: *p*-values for Chi-square test

* Statistically significant difference among groups (*p* < 0.05)

** Statistically significant compared with the negative group

### Infection rates by demographic and risk factors

Protozoal infections were most frequent among participants aged 21–45 years (39.1%) and those > 45 years (36.0%), while *H. pylori* infection was most prevalent in the younger and middle-aged participants (≤ 20 years: 39.5%; 21–45 years: 36.8%); however, neither association reached statistical significance. Infection rates were comparable between sexes. Consumption of tap water was significantly associated with a higher prevalence of both intestinal protozoa (52.6% vs. 18.1%, *p* < 0.001) and *H. pylori* infection (47.4% vs. 22.2%, *p* = 0.001) compared with bottled water. Animal contact and eating outside the home showed no significant association with either infection (Table 4).

**Table 4:** Demographic and behavioral factors associated with intestinal protozoal and *H. pylori* infections among participants

Factors	No. examined	Positive IPI			Positive H. pylori		
		No.	%	*p*	No.	%	*p*
**Age (years)**							0.413
≤ 20	38	11	28.9	0.555	15	39.5	
21–45	87	34	39.1		32	36.8	
> 45	25	9	36.0		6	24.0	
**Gender**							
Male	62	21	33.9	0.648	21	33.9	0.753
Female	88	33	37.5		32	36.4	
**Animal contact**							
No	129	49	38.0	0.209	47	36.4	0.485
Yes	21	5	23.8		6	28.6	
**Eating outdoors**							
No	56	19	33.9	0.683	20	35.7	0.940
Yes	94	35	37.2		33	35.1	
**Water source**							
Tap water	78	41	52.6	**< 0.001** ^*^	37	47.4	0.001^*^
Bottled water	72	13	18.1		16	22.2	

IPI: Intestinal protozoal infection

*p*: *p*-values for Chi-square test

*Statistically significant at *p* < 0.05

### Clinical practice gap

Of the 150 patients referred for *H. pylori* testing, 117 (78%) had not been concurrently referred for parasitological stool examination. Among these, 44 (37.6%) were subsequently found to harbor intestinal protozoal infections.

## Discussion

The true burden of intestinal protozoal infections and *H. pylori* coinfection remains difficult to assess, due to underdiagnosis and limited epidemiological data [21–24]. The present study addressed this gap by identifying intestinal protozoal infections among patients referred for *H. pylori* testing and examining potential associations between the two. Intestinal protozoa were detected in 36.9% of participants, with *Blastocystis* spp. accounting for 50 of the 54 total protozoal detections, followed by *E. dispar* and *E. coli. D. fragilis*,* C. cayetanensis*, and *G. lamblia* were each detected in only four participants. Similar patterns have been reported across other regions of Africa and Europe, where *Blastocystis* spp. consistently predominates among patients with gastrointestinal symptoms [25–28]. Although the pathogenic role of *Blastocystis* remains debated, accumulating evidence links it to chronic gastrointestinal symptoms, gut microbiome disruption, and immune dysregulation. Targeted treatment has been shown to be beneficial in selected symptomatic patients [29]. The non-pathogenic species (*E. dispar* and *E. coli*) co-occurred exclusively with *Blastocystis* and were not detected independently, suggesting that their detection in the present study reflects fecal-oral exposure rather than independent pathological significance, with potential implications for gut microbial composition [30]. With respect to *H. pylori*, 35.3% of patients tested positive. Comparable prevalence rates have been reported in Nigeria (34.8%), Italy (32.6%), and Ethiopia (34.7%) [17, 25, 31], pointing to the enduring global burden of *H. pylori* and its frequent coexistence with enteric protozoa.

Neither intestinal protozoal nor *H. pylori* infections showed significant associations with age or sex, suggesting that exposure to shared risk factors, such as contaminated food or water, inadequate sanitation, and household transmission, may be relatively uniform across demographic groups in this population. Similar findings have been reported elsewhere [32, 33]. Animal contact and eating outside the home were also not significantly associated with either infection. However, drinking water source emerged as a significant risk factor; infection rates for both *H. pylori* and intestinal protozoa were significantly higher among individuals consuming tap water compared with those using bottled water, consistent with findings by Almaw et al. (2024) and Aniekwe et al. (2024), who identified unsafe water and poor sanitation as major determinants of infection [17, 31].

In the present study, intestinal protozoa were more frequent among *H. pylori*-positive patients (39.6%) than among *H. pylori*-negative individuals (34%), though this difference was not statistically significant. This trend may be linked to shared exposure conditions and similar dietary or hygiene practices. Importantly, protozoal infections were significantly more frequent among patients with high *H. pylori* coproantigen levels (54.5% vs. 15.0%), with the strongest association observed for *Blastocystis* spp. (54.5% vs. 5.0%, *p* < 0.001). Previous studies have reported higher rates of intestinal protozoal infections in individuals with elevated *H. pylori* markers, though frequencies vary across populations [34–36]. This association may reflect the higher capacity of protozoa to thrive in the altered gastric or intestinal environment induced by chronic *H. pylori* infection. Specifically, *H. pylori*’s urease activity raises gastric pH [37], and the resulting reduction in gastric acidity possibly create a more permissive environment for protozoan cyst survival [38]. Furthermore, *H. pylori*-related dysbiosis may alter microbial competition and local immune responses, potentially facilitating persistence of organisms such as *Blastocystis* spp. Some reports further suggest more pronounced gastrointestinal symptoms or higher pathogen loads in coinfected patients, pointing to a potential biological interplay between the two infections [39–41]. It should be noted, however, that the cross-sectional design of this study precludes causal inference; the biological mechanisms underlying this association, therefore, remain to be elucidated in future studies.

The clinical overlap between intestinal protozoa and *H. pylori* is notable. In this study, abdominal pain was the most frequent complaint, consistent with earlier reports in which abdominal pain predominated across both protozoal and *H. pylori* infections [42–44]. Diarrhea was the next most common symptom in coinfected patients, whereas *H. pylori* infection alone was characterized by a higher frequency of loss of appetite. Patients with *H. pylori* infection, alone or combined with protozoal infection, were significantly more likely to report symptoms lasting more than three months compared with uninfected participants, whereas coinfection with intestinal protozoa did not appear to further prolong symptom duration. These findings are consistent with the chronic, self-sustaining nature of untreated *H. pylori* infection, in contrast to intestinal protozoal infections, which may be self-limiting in some individuals. Protozoal infections were detected in a proportion of participants in the absence of *H. pylori*, further underscoring the importance of integrating parasitological screening into the differential diagnosis of gastrointestinal complaints.

The present study highlights a substantial diagnostic gap in current clinical practice. The majority of patients not referred for parasitological examination were found to harbor intestinal protozoa, reflecting a marked underuse of stool parasitology. This pattern likely reflects the tendency of overlapping gastrointestinal symptoms to direct clinical attention exclusively toward *H. pylori*, at the expense of a more comprehensive diagnostic evaluation. In resource-limited settings in particular, inadequate diagnostic evaluation may encourage empiric therapy, prolonging illness and obscuring the true etiology. Routine parasitological stool examination alongside *H. pylori* testing, guided by local epidemiological patterns, should therefore be incorporated into diagnostic protocols to improve detection rates, enable targeted treatment, and reduce unnecessary or inappropriate therapy.

Several limitations of this study warrant acknowledgment. *H. pylori* diagnosis was based solely on stool antigen ELISA without endoscopic confirmation, though this method was selected for its non-invasive nature and established diagnostic performance [20]. Only a single stool sample was collected per participant, whereas examination of multiple consecutive samples is recommended to maximize detection sensitivity for intestinal protozoa; this approach was adopted to ensure patient compliance and reflect routine outpatient practice. Furthermore, *E. moshkovskii*, a potentially pathogenic species morphologically identical to *E. histolytica*, could not be excluded, as PCR-based differentiation was not performed [45]. This species has been reported to be relatively uncommon in Egypt [46]. Finally, the single-site recruitment and absence of sample size calculation limit the generalizability of findings.

In conclusion, this study demonstrates a substantial burden of intestinal protozoal infections, predominantly *Blastocystis* spp., among patients undergoing *H. pylori* evaluation, with *Blastocystis* showing a significant association with elevated *H. pylori* antigen concentrations. The high proportion of patients not referred for parasitological examination, among whom protozoal infections were frequently detected, highlights a meaningful diagnostic gap in current clinical practice. Early simultaneous detection of coinfections may enable more targeted therapy and potentially improve clinical outcomes. The present study underscores the need for larger-scale longitudinal studies, and future research should explore the mechanistic pathways through which *H. pylori*-induced mucosal and immune changes might facilitate protozoal colonization.

## Data Availability

All relevant data are included in the manuscript.
